# Large bilateral pulmonary arterial venous malformations treated with embolization and lingulectomy in a patient with hereditary hemorrhagic telangiectasia

**DOI:** 10.1093/jscr/rjab252

**Published:** 2021-06-22

**Authors:** Adele Orovec, Daniel French, Chris Lightfoot, Alison M Wallace

**Affiliations:** Division of Thoracic Surgery, Queen Elizabeth II Health Sciences Centre, Dalhousie University, Halifax, Nova Scotia, Canada; Division of Thoracic Surgery, Queen Elizabeth II Health Sciences Centre, Dalhousie University, Halifax, Nova Scotia, Canada; Department of Diagnostic Imaging, Queen Elizabeth II Health Sciences Centre, Dalhousie University, Halifax, Nova Scotia, Canada; Division of Thoracic Surgery, Queen Elizabeth II Health Sciences Centre, Dalhousie University, Halifax, Nova Scotia, Canada

## Abstract

A 76-year-old woman with hereditary hemorrhagic telangiectasia presented to the emergency department with chest pain. Workup revealed multiple bilateral pulmonary arteriovenous malformations (PAVMs) with large aneurysmal venous outflow. A collaborative approach between interventional radiology and thoracic surgery was used in the treatment of these PAVMs.

## INTRODUCTION

Hereditary hemorrhagic telangiectasia (HHT) is an autosomal dominant genetic condition that interferes with the development of blood vessels. The Curaçao criteria are used in diagnosis and require three of the following: multiple mucocutaneous telangiectasis, spontaneous and recurrent epistaxis, visceral involvement, a family first-degree history of HHT [1]. Pulmonary arteriovenous malformations (PAVMs) are the most common pattern of pulmonary involvement affecting ~15–45% of HHT patients [2]. Management of PAVMs is mainly with percutaneous transcatheter embolization [3]. Major complications of PAVMs include paradoxical systemic embolism, transient ischemic attacks, brain abscess, hemoptysis or hemothorax as well as respiratory symptoms such as dyspnea, cyanosis and clubbing [2].

## CASE REPORT

**Figure 1:**
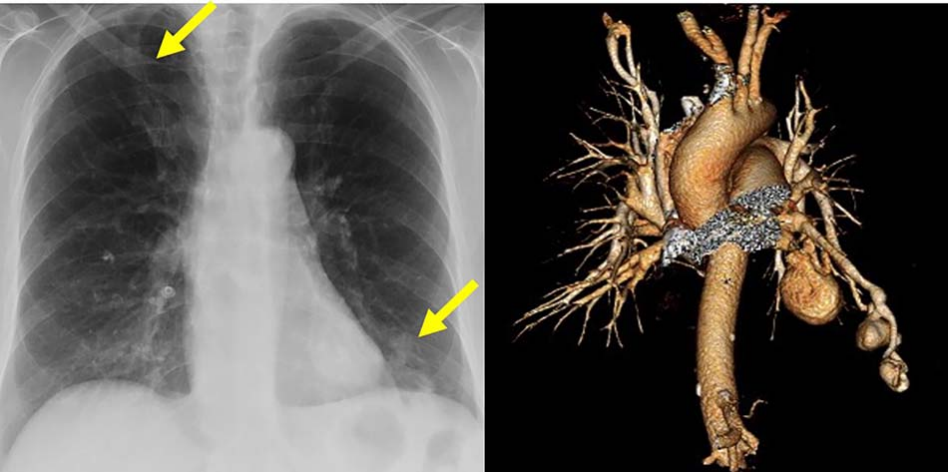
Chest radiograph and computed tomography volume rendered images of multiple aneurysmal PAVMs, including three in lingula and one in right upper lobe. Yellow arrows denote aneurysms.

A 76-year-old woman presented to the emergency department (ED) at a tertiary care hospital complaining of worsening left-sided chest and shoulder pain. The patient underwent diagnostic imaging during her workup in the ED, which included a computed tomography scan of the chest. This revealed three sequential PAVMs off the inferior lingual artery subsegmental branches. The largest had aneurysmal dilation of 3.4 cm, whereas the others measured 2.2 and 1.3 cm in diameter. Additionally, a PAVM in the right upper lobe had aneurysmal dilation of 1.2 cm. All had arterial supply with diameter measuring >3 mm (Fig. 1). Interestingly, the patient had an accessory fissure between the superior and inferior segments of the lingual. No cerebral or gastrointestinal tract arteriovenous malformations were found in workup.

The patient was diagnosed with HHT as a child. Up until her presentation to the ED her only manifestations were frequent nosebleeds starting when she was a teenager and a few skin telangiectasias. The patient had not had ongoing screening for PAVMs, cerebral AVMs or other abnormalities related to HHT.

**Figure 2:**
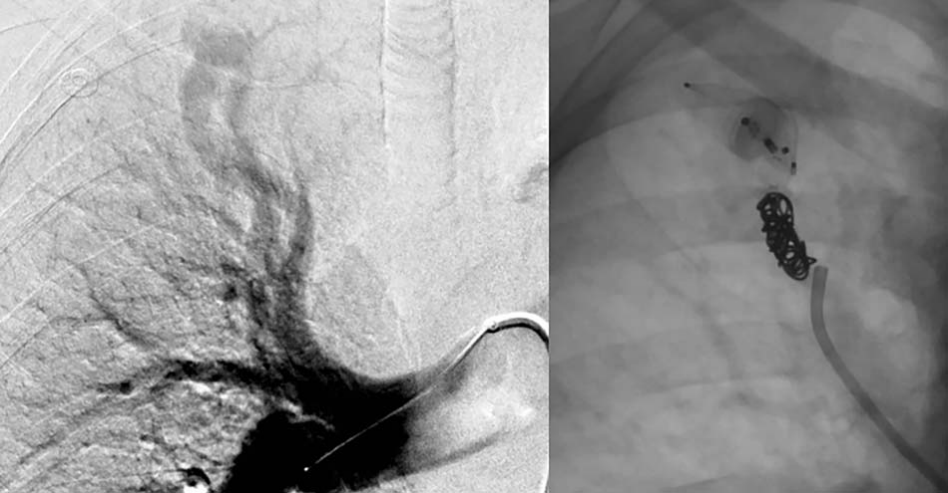
Embolization of right upper lobe PAVM with second and fourth generation Amplatzer™ Vascular Plugs and Nester® Embolization Coils.

The patient’s paternal grandmother, father and uncle had HHT. Her father and uncle died of complications related to HHT at 57 and 72, respectively. The patient has two adult sons, and they each have two children. One son has been diagnosed with HHT along with his daughter; however, her twin brother does not have HHT. The patient’s other son also has HHT along with one of his two children (Fig. 2).

**Figure 3:**
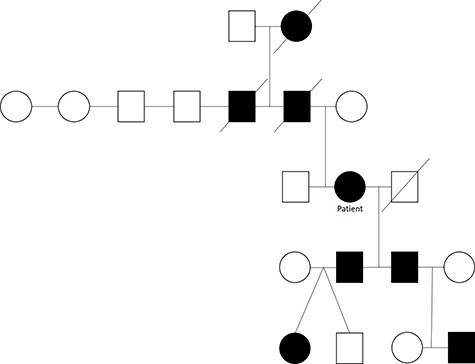
A pedigree of a family with HHT a rare autosomal dominant genetic disorder that leads to abnormal blood vessel formation in the skin, mucous membranes as well as the lungs, liver and brain.

The treatment plan consisted of embolization of the isolated simple right upper lobe PAVM followed by surgical resection of the large left-sided aneurysmal PAVMs. A surgical approach dealing with the three left-sided PAVMs was felt to be most appropriate, given that all arose from inferior lingual artery and had very large aneurysm dilation. Two days after successful embolization of the right upper lobe PAVM with second and fourth generation Amplatzer™ Vascular Plugs (Abbott Cardiovascular, Plymouth MN) and Nester® Embolization Coils (Cook Medical, Bloomington, IN), the patient underwent a left thoracotomy and lingulectomy for resection of the left-sided AVMs (Fig. 3). Before proceeding with the lingulectomy, we obtained proximal and distal vascular control with a Rummel tourniquet around the left main pulmonary artery and vessel loops around the superior and inferior pulmonary veins. The lingulectomy was performed in the standard fashion. The patient’s postoperative course was uncomplicated.

**Figure 4:**
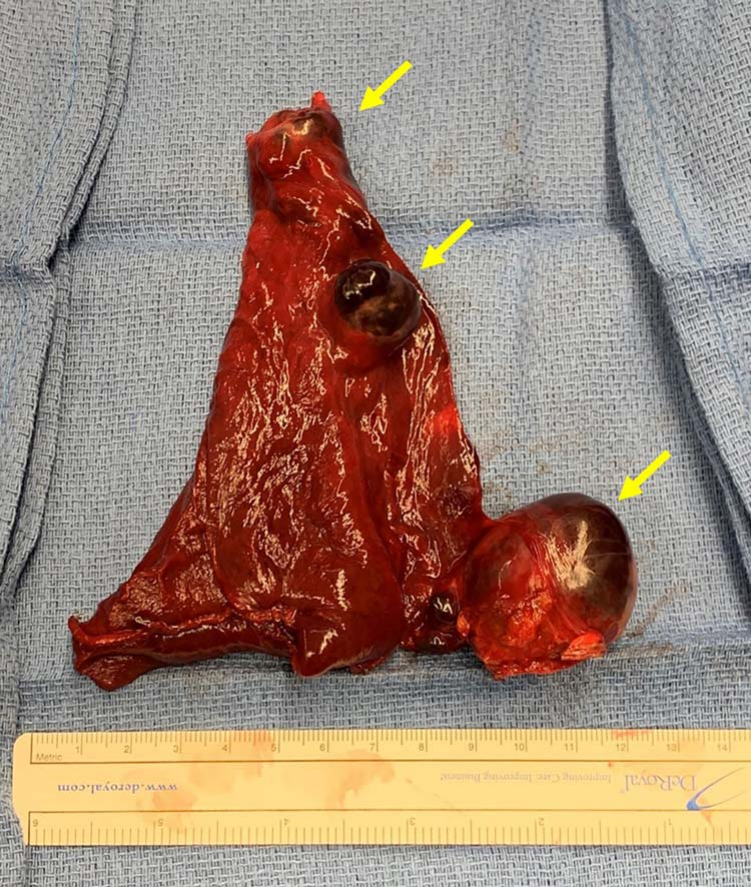
Lingulectomy specimen showing three sequential PAVMs off the inferior lingual artery subsegmental branches. Yellow arrows denote aneurysms.

## DISCUSSION

This case presents four large PAVMs in the setting of HHT. At least 33% of patients with HHT have a single PAVM and at least 50% have multiple PAVMs [4]. Historically, surgery was the mainstay of treatment for PAVMs. Although percutaneous image-guided embolization is now often first-line treatment, there remains a role for surgical management in this complex disease [3]. A VATS approach was used initially in this case but after assessment with the thoracoscope we converted to open to obtain proximal and distal vascular control before dissection around the large and friable vessels. A collaborative approach between interventional radiology and thoracic surgery at our institution optimized the management of this patient with HHT and multiple large aneurysmal PAVMs.

## CONFLICT OF INTEREST STATEMENT

None declared.

## References

[1] Shovlin CL , Buscarini E, Kjeldsen AD, Mager HJ, Sabba C, Droege F, et al. European Reference Network for Rare Vascular Diseases (VASCERN) outcome measures for hereditary haemorrhagic telangiectasia (HHT). Orphanet J Rare Dis 2018;13:1–5. 30111344 10.1186/s13023-018-0850-2 PMC6094583

[2] Donaldson JW , McKeever TM, Hall IP, Hubbard RB, Fogarty AW. Complications and mortality in hereditary hemorrhagic telangiectasia. Neurology 2015;84:1886–93. 25862798 10.1212/WNL.0000000000001538 PMC4433463

[3] Lacombe P , Lacout A, Marcy PY, Binsse S, Sellier J, Bensalah M, et al. Diagnosis and treatment of pulmonary arteriovenous malformations in hereditary hemorrhagic telangiectasia: an overview. Diagn Interv Imaging 2013;94:835–48. 23763987 10.1016/j.diii.2013.03.014

[4] Suchin CR , Whitman GJ, Chew FS. Pulmonary arteriovenous malformation. Am J Roentgenol 1996;167:648. 8751671 10.2214/ajr.167.3.8751671

